# Unsupervised Clustering Successfully Predicts Prognosis in NSCLC Brain Metastasis Cohorts

**DOI:** 10.3390/diagnostics15141747

**Published:** 2025-07-10

**Authors:** Emre Uysal, Gorkem Durak, Ayse Kotek Sedef, Ulas Bagci, Tanju Berber, Necla Gurdal, Berna Akkus Yildirim

**Affiliations:** 1 Department of Radiation Oncology, University of Health Science, Prof. Dr. Cemil Tascioglu City Hospital, Istanbul 34390, Turkeyberna.yildirim@sbu.edu.tr (B.A.Y.); 2 Machine & Hybrid Intelligence Lab, Department of Radiology, Northwestern University, Chicago, IL 60611, USA; gorkem.durak@northwestern.edu; 3 Department of Radiation Oncology, Dr. Ersin Arslan Research and Training Hospital, Gaziantep 27000, Turkey

**Keywords:** brain metastases, non-small-cell lung cancer, prognostic factors, unsupervised learning, clustering

## Abstract

**Background/Objectives**: Current developments in computer-aided systems rely heavily on complex and computationally intensive algorithms. However, even a simple approach can offer a promising solution to reduce the burden on clinicians. Addressing this, we aim to employ unsupervised cluster analysis to identify prognostic subgroups of non-small-cell lung cancer (NSCLC) patients with brain metastasis (BM). Simple-yet-effective algorithms designed to identify similar group characteristics will assist clinicians in categorizing patients effectively. **Methods**: We retrospectively collected data from 95 NSCLC patients with BM treated at two oncology centers. To identify clinically distinct subgroups, two types of unsupervised clustering methods—two-step clustering (TSC) and hierarchical cluster analysis (HCA)—were applied to the baseline clinical data. Patients were categorized into prognostic classes according to the Diagnosis-Specific Graded Prognostic Assessment (DS-GPA). Survival curves for the clusters and DS-GPA classes were generated using Kaplan–Meier analysis, and the differences were assessed with the log-rank test. The discriminative ability of three categorical variables on survival was compared using the concordance index (C-index). **Results**: The mean age of the patients was 61.8 ± 0.9 years, and the majority (77.9%) were men. Extracranial metastasis was present in 71.6% of the patients, with most (63.2%) having a single BM. The DS-GPA classification significantly divided the patients into prognostic classes (*p* < 0.001). Furthermore, statistical significance was observed between clusters created by TSC (*p* < 0.001) and HCA (*p* < 0.001). HCA showed the highest discriminatory power (C-index = 0.721), followed by the DS-GPA (C-index = 0.709) and TSC (C-index = 0.650). **Conclusions**: Our findings demonstrated that the TSC and HCA models were comparable in prognostic performance to the DS-GPA index in NSCLC patients with BM. These results suggest that unsupervised clustering may offer a data-driven perspective on patient stratification, though further validation is needed to clarify its role in prognostic modeling.

## 1. Introduction

Lung cancer is not only the most common cause of brain metastases (BM) but is also the leading cause of cancer-related death worldwide [1,2]. Non-small-cell lung cancer (NSCLC), which accounts for up to 80% of lung cancers, develops BM in approximately 30% to 50% of cases [2]. Approximately 20% of NSCLC patients present with synchronous BM, most of which involve single sites of distant metastasis [3,4]. BM significantly decreases overall survival (OS) and quality of life in patients with NSCLC [5,6,7]. Recently, BM was regarded as a single disease, and treatment options for BM were limited, leading to extremely poor survival rates of less than six months. Nowadays, the life expectancy of patients has increased thanks to the development of stereotactic radiosurgery and systemic therapies. In addition, advances in diagnostic methods, such as magnetic resonance imaging (MRI), have significantly improved the detection of small BMs [8].

Several prognostic scoring systems have been developed to predict expected survival times and guide clinicians in disease management. Gaspar et al. demonstrated the first prognostic classification for patients with BM in 1997 [9]. This classification, known as recursive partitioning analysis (RPA), involved dividing patients into three categories based on the prognostic factors: Karnofsky performance status (KPS), primary control status, age, and presence of extracranial metastasis (ECM). In 2008, Sperduto et al. introduced the Grading Prognostic Assessment (GPA), which is at least as prognostic as RPA and more quantitative [10]. However, these prognostic scoring systems were developed without considering the primary diagnosis. Since prognostic factors depend on the primary diagnosis, Sperduto et al. identified and validated the Diagnosis-Specific GPA (DS-GPA) in 2012 [11,12]. Additionally, Sperduto et al. reported the first update of the prognostic index using molecular biomarkers for NSCLC patients in 2017 [13]. Four prognostic classes were established in the updated DS-GPA index (Lung-molGPA) based on the total score obtained from five prognostic factors. Our previous study showed that the DS-GPA was a useful and effective prognostic biomarker in our cohort [14].

After the significant breakthrough of artificial intelligence in 2012 with deep convolutional neural networks, researchers have focused on developing improved prognostic models using deep learning. Several studies have reported promising machine learning models that predict OS better than traditional models for patients with BM [15,16]. However, the models demonstrated in the studies employed supervised deep learning algorithms, which require large-scale, well-curated data that is often difficult to obtain, leading to overfitting and generalization issues. Unsupervised learning algorithms may better address this problem, particularly when data is limited and curation poses challenges. Recently, unsupervised learning methods have gained attention in prognostication research, with several studies showing more favorable outcomes than traditional staging methods [17,18,19,20,21]. Although the number of such studies remains limited, unsupervised learning methods have also been applied in various cancer types, including breast and lung cancers, to identify clinically meaningful subgroups [22,23,24,25].

However, to the best of our knowledge, no study has explored unsupervised clustering for prognostic classification in the specific context of NSCLC patients with brain metastases. In this study, we aimed to explore the ability of unsupervised clustering methods to identify prognostic subgroups among these patients in comparison to the established Lung-molGPA index. We hypothesized that unsupervised clustering would stratify patients into subgroups with significantly different survival outcomes, potentially outperforming existing prognostic models.

## 2. Materials and Methods

### 2.1. Patients

The study was designed using retrospective data obtained from two oncology centers. A total of 95 NSCLC patients with BM, who were admitted to the centers between 2015 and 2021, were included in the study. Demographic features, prognostic variables, and OS times of the patients whose NSCLC diagnosis was confirmed by biopsy were collected from the hospital archives. Patients’ age, Karnofsky performance status, extracranial metastasis, number of brain metastases, and gene status, which are prognostic factors according to the DS-GPA, were reviewed (Table 1) [13]. BMs were detected using thin-slice (1 mm) contrast-enhanced MRI. The presence of ECM was examined using contrast-enhanced computed tomography and/or positron emission tomography/computed tomography.

Patients were included if complete baseline data were available for key prognostic variables and survival outcomes. Cases with missing data on these variables were excluded from the analysis. The dataset was derived from a previous study investigating the prognostic value of GPA scores in NSCLC patients with brain metastases [14]. The study received approval from the Ethics Committee of Prof. Dr. Cemil Tascioglu City Hospital, and informed consent was waived due to its retrospective design.

### 2.2. Unsupervised Cluster Analysis

Prognostic factors for OS included age, KPS, presence of ECM, number of BM, and molecular subtypes according to Lung-molGPA. Since all patients’ EGFR and ALK statuses were either negative or unknown (corresponding to a score of 0 in the DS-GPA), these variables were excluded from the clustering analysis. These factors were utilized to identify natural clusters of similar cases, without any external guidance or human intervention. Therefore, two-step clustering (TSC) and hierarchical cluster analysis (HCA) were employed to distinguish the patients. TSC and HCA were selected due to their suitability for datasets with mixed variable types (categorical and continuous) and small-to-moderate sample sizes. Both methods support automatic estimation of the number of clusters, making them well-suited for exploratory analyses without requiring prior assumptions.

Error optimization is essential in both unsupervised and supervised learning. While labeled inputs are crucial for calculating the error between the expected and actual output values in supervised learning, the error in unsupervised learning is minimized by comparing the unlabeled inputs [26].

For TSC, we used a two-stage process. In the pre-clustering stage, a clustering feature (CF) tree was built using the log-likelihood distance measure, which is suitable for mixed-type data. In the second stage, hierarchical clustering was applied to these pre-clusters to derive the final cluster solution. The number of clusters was automatically determined using the Bayesian Information Criterion (BIC), which balances model fit with complexity, and the algorithm identified four optimal clusters [27].

For HCA, Euclidean distance was computed between all cases after standardizing continuous variables and numerically encoding categorical variables. Ward’s linkage method was used, as it tends to create compact and homogeneous clusters, which is appropriate for detecting subtle group structures in moderate-sized datasets. The number of clusters was determined by visual inspection of the dendrogram, where a natural break in the tree structure suggested a four-cluster solution, consistent with the TSC results. Internal cluster validation was performed using silhouette analysis based on appropriate distance metrics for mixed-type variables. In addition, a principal component analysis (PCA)-based plot was generated to illustrate the separation of clusters in two-dimensional space.

### 2.3. Statistics

Descriptive data were presented as means ± standard error (SE) for continuous variables and as numbers (%) for categorical variables. Survival curves were generated using Kaplan–Meier analysis, and the log-rank test was utilized to compare the OS of the patients. The concordance index (C-index) was calculated in R (version 4.5.1) to assess the discriminative performance of each classification method. Cox’s proportional hazards regression was also performed to estimate hazard ratios (HRs) with 95% confidence intervals (CIs) for each subgroup within the DS-GPA, TSC, and HCA classifications. Differences in categorical variables between clusters were analyzed using the chi-square test, and differences in continuous variables were assessed with the Kruskal–Wallis test. Statistical analyses were conducted using IBM SPSS Statistics for Windows, version 26 (IBM Corp., Armonk, NY, USA), with a statistical significance level set at *p* < 0.05.

## 3. Results

The mean age of the patients was 61.8 ± 0.9, and 74 (77.9%) were men. The median OS for the patients was 8 months (95% CI, 6.2–9.8). All NSCLC patients had BM, ranging from 1 to 11. While 60 (63.2%) patients had a single BM, 82 (86.3%) patients had fewer than four BMs. Most of the patients (71.6%) were able to carry on regular activities with a KPS of 80 and 90 at the time of BM diagnosis. Specifically, 4 patients (4.2%) had a KPS of 60, 23 (24.2%) had a KPS of 70, 36 (37.9%) had a KPS of 80, and 32 (33.7%) had a KPS of 100. ECM was present in 68 (71.6%) patients.

**Figure 1:**
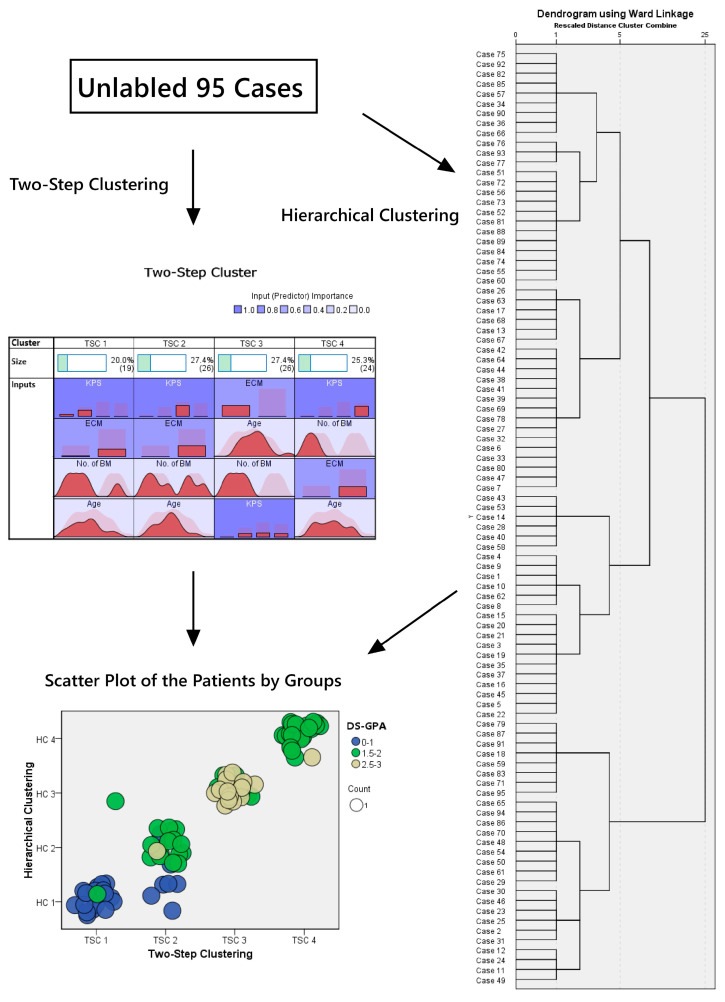
Clustering algorithms and the distribution of the patients by groups.

The patients were divided into three categories, and the sum of the scores was calculated based on the DS-GPA index, which was previously defined as four prognostic classes. Since all patients’ EGFR and ALK statuses were either negative or unknown (corresponding to a score of 0 in the DS-GPA), these variables were excluded from the cluster analyses. Additionally, the patients were separated into four clusters using TSC and HCA. The distribution of patients using the three classification methods is illustrated in Figure 1 with a scatter plot. Furthermore, the characteristics of the prognostic factors in the classes are provided in Table 2. A comparative visualization of clinical variables across the TSC and HCA clusters is also presented in Supplementary Figure S1. The silhouette score was 0.45 for TSC and 0.42 for HCA, indicating moderate internal consistency for both models. The PCA-based plot (Supplementary Figure S2) visually demonstrates the spatial distribution and partial separation of clusters.

Each of the four clusters demonstrated distinct clinical profiles, supported by statistical comparisons. Across both clustering methods, KPS significantly differed between clusters (TSC and HCA: *p* < 0.001), with better prognosis clusters having higher KPS scores. ECM was present exclusively in Cluster 3 in both models and showed a significant difference (TSC and HCA: *p* < 0.001). The number of brain metastases also varied significantly (TSC: *p* = 0.031; HCA: *p* = 0.001), with the poorest prognosis cluster exhibiting the highest burden. Age did not differ significantly in TSC-based clustering (*p* = 0.113) but reached statistical significance in HCA (*p* = 0.045), primarily due to a younger median age in the poorest prognosis group.

**Figure 2:**
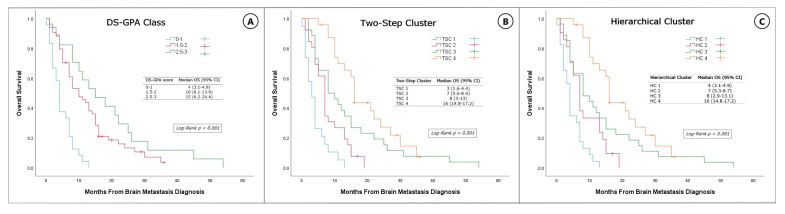
Kaplan–Meier survival curves of non-small-cell lung cancer patients with brain metastasis, grouped by DS-GPA (**A**), two-step cluster analysis (**B**), and hierarchical cluster analysis (**C**). The table included in the figure displays the median OS and 95% CI for each group.

There was a statistically significant difference in OS across the DS-GPA categories (*p* < 0.001). Similarly, both the TSC- and HCA-derived clusters showed a strong prognostic association with OS (*p* < 0.001 for both, log-rank test). These findings highlight the clinical relevance of the unsupervised clustering approaches. OS curves and median survival estimates for each group are presented in Figure 2. HCA showed the highest discriminatory power (C-index = 0.721), followed by the DS-GPA (C-index = 0.709) and TSC (C-index = 0.650)

HRs with a 95% CI were calculated for each subgroup within the DS-GPA, TSC, and HCA classifications (Table 3). All three models showed significant differences in OS between subgroups (*p* < 0.001).

## 4. Discussion

In this study, we evaluated the potential of unsupervised clustering for prognostic classification in NSCLC patients with BM. Prognostic factors for OS included age, KPS, ECM presence, BM number, and molecular subtypes according to Lung-molGPA. The classes based on the DS-GPA index significantly differentiated the patients regarding OS (*p* < 0.001). Additionally, we found a highly significant difference in OS between the classes of patients clustered through unsupervised cluster analyses (*p* < 0.001 for TSC, and *p* < 0.001 for HCA). Among the three approaches, the highest discriminatory performance was observed for HCA (C-index = 0.721), followed by the DS-GPA (0.709) and TSC (0.650). Although the C-index values suggest improved stratification with clustering methods, the absolute differences between models were clinically modest. Therefore, these results should be interpreted with caution, and independent, larger-scale validation is warranted.

Although drug transport into the brain is limited, advancements in systemic treatments, such as targeted therapy for metastatic disease, have improved the OS of NSCLC patients. Consequently, the incidence of patients diagnosed with BM has increased as life expectancy has grown. Moreover, with the development of stereotactic radiotherapy techniques, high-dose radiation can be precisely delivered to the tumor while quickly reducing the dose to the surrounding healthy tissue. For these reasons, efforts to predict the survival of patients with BM have increased to determine the optimal treatment for these patients [28]. Several randomized controlled trials indicate that stereotactic radiotherapy reduces neurocognitive deterioration and enhances quality of life in selected patients without compromising local control and survival when compared to whole-brain radiotherapy [29,30,31]. The recent guide, reported in cooperation with the American Society of Clinical Oncology, the Society for Neuro-Oncology, and the American Society for Radiation Oncology, suggests that stereotactic radiotherapy should be offered to patients who have a good performance status and a limited number of BMs [32].

In previously published research, various prognostic classifications were developed based on age, KPS, presence of ECM, number of BMs, maximum tumor volume, and primary tumor control [8,9,10,33]. Different scoring was used based on the weight of prognostic factors in classifications that included similar factors. In 2018, Gao et al. compared four common indexes: RPA, GPA, Score Index for Radiosurgery (SIR), and Basic Score for Brain Metastases (BSBM) in NSCLC patients with BM after stereotactic radiotherapy [34]. They reported that all four indexes significantly predict OS, with BSBM being a better prognostic index than the others. BSBM incorporates three predictors: KPS, control of the primary tumor, and ECM, while SIR includes age, the number of BMs, and larger lesion volume but does not consider ECM [8,30]. Moreover, prognostic classifications for predicting OS in patients have frequently been updated with the advancement of BM management [10,11,12,13].

Machine learning algorithms have surpassed traditional statistics over the last decade, particularly in predicting cancer outcomes. While independent prognostic factors are essential for predicting OS, traditional methods may prove inadequate for evaluating high-dimensional data. Characteristics of patients and diseases that are not recognized as independent prognostic factors are often excluded from the multivariable prognostic model, causing interactions among multiple factors to be overlooked. Nevertheless, few machine-learning-based studies addressing prognosis in disease-specific BM are present in the literature. Huang et al. have explored the optimal prognostic index using machine learning for BM without distinguishing between primary diseases [16]. They enrolled seven hundred patients and selected seven features to generate a prognostic model. To achieve the best prognostic model, seven supervised machine learning algorithms were utilized for classification, along with four feature selection methods. In this study, the MIRSPSO feature selection method (mutual information and rough set of particle swarm optimization) achieved the highest area under the curve (AUC) across all machine learning methods. The random forest classifier demonstrated the best prognostic performance, achieving an AUC of 0.978. Additionally, they showed that BSBM has greater accuracy than RPA, GPA, and SIR and that the machine learning method outperforms conventional statistical methods.

Two large-sample studies compared supervised machine learning methods to predict OS in breast cancer patients with BM. In a recently published study involving 1933 patients, Li et al. generated an XGBoost prediction model (eXtreme Gradient Boosting), an ensemble learning method utilizing decision trees. They reported that the XGBoost model outperformed five supervised machine learning models [35]. In another study, Bice et al. compared the predictive performances of three methods in OS, involving 1673 patients [15]. They stated that DeepSurv (a deep learning algorithm structured similarly to the Cox regression model and available for public use) and random forest models trained with 27 covariates outperformed the Cox regression model [36].

To our knowledge, no study in the literature has investigated the potential of unsupervised learning in categorizing NSCLC patients with BM. Therefore, it is not possible to compare our results directly with the existing literature. Clustering methods are commonly used to identify homogeneous subclasses within datasets. This algorithm utilizes the interactions among various data to reveal the underlying structures of patients who share common characteristics, such as similar diseases, outcomes, or other conditions. In our study, we found a highly significant difference in OS between the classes of patients clustered by unsupervised learning based on patient similarities.

This study offers a novel perspective on prognostic modeling by exploring how unsupervised learning can be used to identify clinically meaningful subgroups in a well-defined oncologic population. While most prior research has focused either on rule-based indices or supervised algorithms, our approach investigates whether patient stratification can emerge organically from routine clinical variables—without the need for predefined scores or outcome labels. By comparing two unsupervised methods and aligning their output with survival outcomes, we provide an alternative lens for understanding patient heterogeneity in NSCLC with brain metastases.

The usefulness of this approach lies not only in its conceptual shift but also in its adaptability to real-world clinical datasets. In contrast to recent supervised learning models that require large, curated datasets and technical infrastructure [15,16,35], our method operates on basic clinical variables and does not depend on outcome annotation. This flexibility is particularly relevant in settings where data complexity or scale may hinder traditional modeling efforts. Although the model is not yet validated for clinical use, its capacity to uncover prognostically distinct subgroups suggests potential value as a complementary tool in future stratification frameworks.

From our perspective, achieving the results was not surprising because we utilized previously determined prognostic factors. However, despite the similar prognostic significance among the models (*p* < 0.001 for all three classifications), a few patient classes differed based on the methods used. These findings suggest that unsupervised clustering methods—particularly HCA—may offer complementary prognostic value compared to traditional classifications. Unsupervised learning, by leveraging high-dimensional data analysis and pattern discovery, has the potential to improve stratification in selected patient populations. Furthermore, multi-omics data is necessary to elucidate the underlying structures of patients with common characteristics such as similar diseases, outcomes, or situations. Although supervised learning is generally preferred in machine-learning-based studies, the hope is that unsupervised learning may prove more efficient [26].

Confounding variables pose a significant challenge to unsupervised clustering and even to supervised classification, where the predicted variables are labeled, as they can mislead feature selection algorithms [37]. However, feature selection algorithms were not applied in the study because the selected features were based on previously demonstrated prognostic factors. We will collect multi-omic data to enhance the performance of unsupervised learning by leveraging the interactions among various features.

The data-driven subgroups revealed by unsupervised clustering correspond to clinically recognizable phenotypes that can inform treatment selection and follow-up intensity. Patients clustering together with favorable performance status and limited intracranial disease may be suitable for aggressive local therapies or intensified systemic approaches, whereas those grouped by poor performance status and high tumor burden are more likely to benefit from symptom-directed palliation. Importantly, the method isolated an ECM-positive subgroup that is not explicitly captured by traditional scores, signaling a need for closer systemic surveillance or earlier therapy escalation. Beyond these immediate clinical insights, the unsupervised framework offers practical advantages over fixed-weight indices such as DS-GPA: it accommodates complex, non-linear interactions among variables, adapts seamlessly when novel biomarkers (e.g., radiomic or multi-omic features) are added, and achieves this without prespecifying cut-off values. As new data modalities are integrated, this flexible approach is well positioned to refine prognostic stratification and support truly personalized care.

This study has some limitations. Unexpected biases stemming from the retrospective design may have influenced our results. Although our findings were consistent with the literature, the study’s sample size was relatively small. Because of the limited sample size, neither external validation nor internal cross-validation could be performed; future multi-center studies are required to confirm these findings. Moreover, since EGFR and ALK mutation statuses were either negative or unknown in all patients, these molecular variables were excluded from the clustering process. As a result, the prognostic classifications did not account for the impact of molecular and histological subtypes, which may limit the generalizability of our findings to molecularly stratified populations. Given these limitations, the current model is not suitable for clinical application. Nonetheless, the results may provide an early indication of the potential value of unsupervised clustering methods for future risk models, pending further validation.

## 5. Conclusions

In conclusion, we evaluated the prognostic performance of unsupervised clustering using previously defined clinical factors in NSCLC patients with brain metastases. Our findings showed that both the TSC and HCA models produced stratifications that aligned with overall survival and were comparable to the DS-GPA index. These results suggest that unsupervised methods can uncover meaningful patterns within routine clinical data. Further studies involving prospective validation and methodological refinement are needed to better understand the scope and limitations of such approaches.

## Figures and Tables

**Table 1:** Updated DS-GPA (Lung-molGPA) scoring.

Prognostic Factor	GPA Scoring Criteria		
	0	0.5	1.0
Age, years	≥70	<70	NA
KPS	≤70	80	90–100
ECM	Present		Absent
Brain metastases, No.	>4	1–4	NA
Gene status	*EGFR* neg/unk and *ALK* neg/unk	NA	*EGFR* pos or *ALK* pos

Abbreviations: DS, diagnosis-specific; ECM, extracranial metastases; GPA, graded prognostic assessment; KPS, Karnofsky performance status; NA, not applicable; neg/unk, negative or unknown; pos, positive.

**Table 2:** Distribution of the prognostic factors in the groups.

			DS−GPA			Two-Step Clustering				Hierarchical Clustering			
			0–1 *n* = 24	1.5–2 *n* = 54	2.5–3 *n* = 17	TSC 1 *n* = 19	TSC 2 *n* = 26	TSC 3 *n* = 26	TSC 4 *n* = 24	HC 1 *n* = 23	HC 2 *n* = 21	HC 3 *n* = 27	HC 4 *n* = 24
Age		Mean	60.3	62	63.4	59.1	61.4	65.1	60.8	58.3	62.8	65.0	60.8
		SE	2.1	1.2	1.3	2.3	1.6	1.6	1.8	2.0	1.7	1.5	1.8
KPS	60	*n*	3	1	−	4	−	−	−	3	−	1	−
		%	75	25		100				75		25	
	70	*n*	14	8	1	15	−	8	−	15	−	8	−
		%	60.9	34.8	4.3	65.2		34.8		65.2		34.8	
	80	*n*	7	22	7	−	26	10	−	5	21	10	−
		%	19.4	61.1	19.4		72.2	27.8		13.9	58.3	27.8	
	90	*n*	−	23	9	−	−	8	24	−	−	8	24
		%		71.9	28.1			25.0	75.0			25.0	75.0
ECM		*n*	24	42	2	18	26	−	24	23	21	−	24
		%	35.3	61.8	2.9	26.5	38.2		35.3	33.8	30.9		35.3
No. of BM		Mean	3.7	1.6	1.6	2.8	2.8	1.7	1.5	3.9	1.6	1.7	1.5
		SE	0.6	0.1	0.2	0.5	0.5	0.2	0.2	0.6	0.2	0.2	0.2

Abbreviations: BM, brain metastasis; DS, diagnosis-specific; ECM, extracranial metastases; GPA, graded prognostic assessment; HC, hierarchical cluster; KPS, Karnofsky performance status; TSC, two-step cluster.

**Table 3:** Cox’s univariate proportional hazards regression analysis for overall survival.

Prognostic Classes	Univariate Regression Analysis		
	HR	95% CI	*p*
DS-GPA			
0–1	ref		<0.001
1.5–2	0.297	0.174–0.508	<0.001
2.5–3	0.195	0.096–0.396	<0.001
TSC			
TSC 1	ref		<0.001
TSC 2	0.434	0.235–0.800	0.007
TSC 3	0.214	0.110–0.416	<0.001
TSC 4	0.130	0.065-0.263	< 0.001
HCA			
HCA 1	ref		<0.001
HCA 2	0.457	0.246–0.848	0.013
HCA 3	0.257	0.137–0.482	<0.001
HCA 4	0.150	0.076–0.295	<0.001

Abbreviations: DS, diagnosis-specific; GPA, graded prognostic assessment; TSC, two-step clustering; HCA, hierarchical cluster analysis; HR, hazard ratio; CI, confidence interval.

## Data Availability

The data presented in this study are available on request from the corresponding author due to privacy and ethical restrictions.

## Supplementary Materials

The following supporting information can be downloaded at: https://www.mdpi.com/article/10.3390/diagnostics15141747/s1, Figure S1: Comparison of cluster characteristics using both unsupervised methods. Distributions of age, BM number, KPS, and ECM-positive rates are shown across TSC clusters (x-axis), color-coded by HCA membership. This visualization highlights similarities and differences in patient stratification between TSC and HCA. Figure S2: PCA-based visu-alization of cluster separation for (A) Two-Step Clustering (TSC) and (B) Hierarchical Cluster Analysis (HCA). The plot shows the distribution of patients in the two-dimensional space defined by the first two principal components derived from age, number of brain metastases, KPS, and ECM status. Color represents cluster membership. While some overlap is observed, both methods demonstrate distinguishable group structures.

## Abbreviations

The following abbreviations are used in this manuscript:

NSCLC	Non-small-cell lung cancer
BM	Brain metastasis
TSC	Two-step clustering
HCA	Hierarchical cluster analysis
DS-GPA	Diagnosis-Specific Graded Prognostic Assessment
OS	Overall survival
RPA	Recursive partitioning analysis
KPS	Karnofsky performance status
ECM	Extracranial metastasis
SE	Standard error
SIR	Score Index for Radiosurgery
BSBM	Basic Score for Brain Metastases
PCA	Principal component analysis
HR	Hazard ratio
CI	Confidence interval
AUC	Area under the curve
MISPRO	Mutual information and rough set of particle swarm optimization
XGBoost	eXtreme Gradient Boosting
