# Supplementary material for: Unsupervised Clustering Successfully Predicts Prognosis in NSCLC Brain Metastasis Cohorts
Source: Diagnostics (Basel). 2025 Jul 10;15(14):1747. doi: 10.3390/diagnostics15141747 (PMC12293156; doi:10.3390/diagnostics15141747)
Supplement: Supplementary file 1 [file diagnostics-15-01747-s001.zip › diagnostics-3660235-supplementary.pdf]

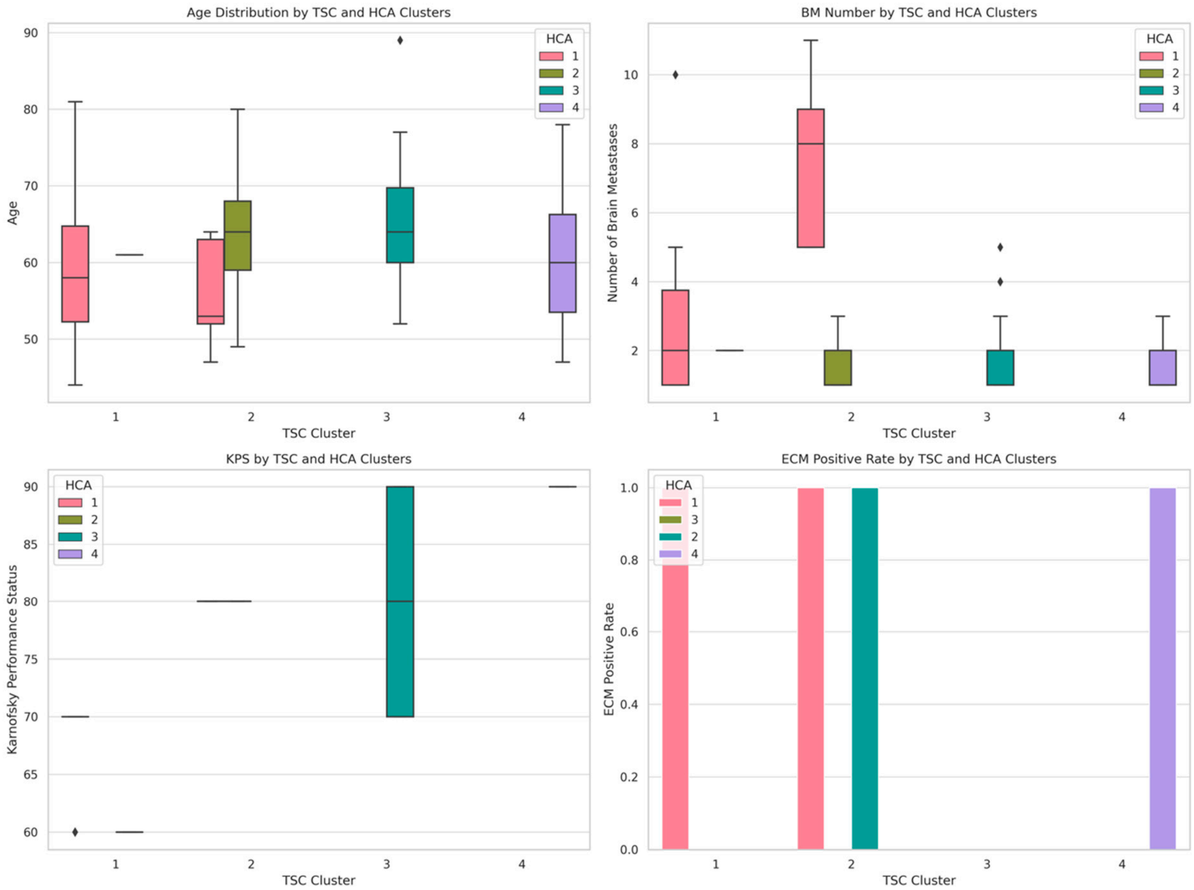

Supplementary Figure S1. Comparison of cluster characteristics using both unsupervised methods. Distributions of age, BM number, KPS, and ECM-positive rates are shown across TSC clusters (x-axis), color-coded by HCA membership. This visualization highlights similarities and differences in patient stratification between TSC and HCA.

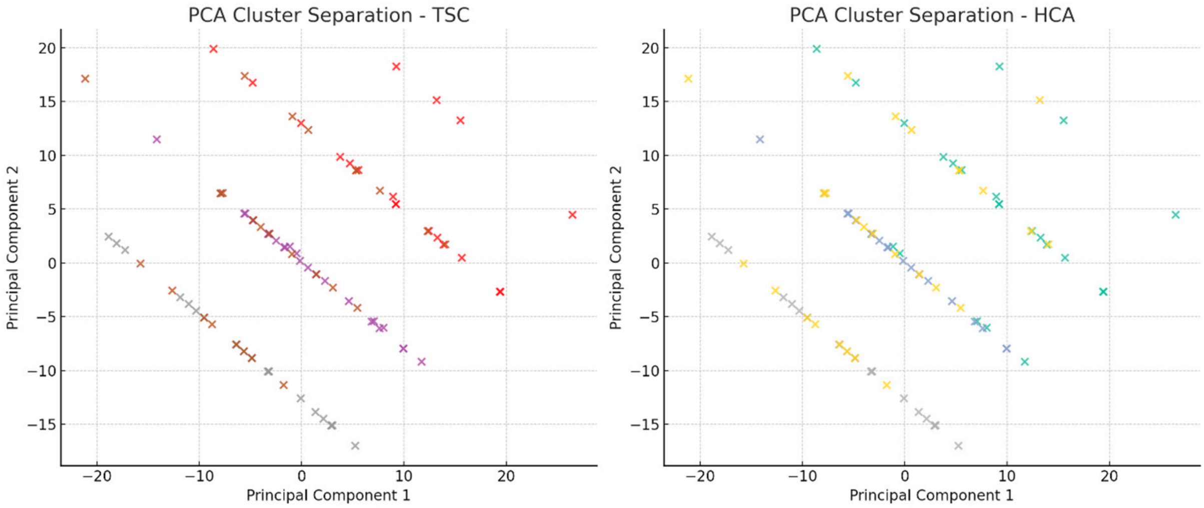

Supplemantary Figure S2. PCA-based visualization of cluster separation for (A) Two-Step Clustering (TSC) and (B) Hierarchical Cluster Analysis (HCA). The plot shows the distribution of patients in the two-dimensional space defined by the first two principal components derived from age, number of brain metastases, KPS, and ECM status. Color represents cluster membership. While some overlap is observed, both methods demonstrate distinguishable group structures.
